# Perianal Paget Disease: Different Entities With the Same Name

**DOI:** 10.7759/cureus.15161

**Published:** 2021-05-22

**Authors:** Marisa D Santos, Filomena Soares, José M Presa-Fernandes, Donzília S Silva

**Affiliations:** 1 Colorectal Surgery, Instituto de Ciências Biomédicas Abel Salazar (ICBAS) - University of Porto, Porto, PRT; 2 Colorectal Surgery, Centro Hospitalar Universitário do Porto, Porto, PRT; 3 Colorectal Surgery, Centro Hospitalar Tâmega e Sousa, Penafiel, PRT; 4 Breast Surgery, Centro Hospitalar Universitário do Porto, Porto, PRT; 5 Hepatobiliary and Pancreatic Surgery, Centro Hospitalar Universitário do Porto, Porto, PRT

**Keywords:** perianal paget’s disease, extramammary paget’s disease, surgery in perianal paget´s disease

## Abstract

Extramammary Paget disease (EMPD) is a rare form of cancer that involves skin areas rich in apocrine glands. The common sites of occurrence include the vulva, perianal region, perineum, and axilla. Perianal Paget disease (PPD) is a subset of EMPD, which arises in the perianal skin. The disease commonly presents with a thickened plaque-like lesion with erythema or white scaly appearance. It is generally classified into two categories based on the origin of the tumor cells: (1) primary PPD if the tumor arises from intraepidermal cells and (2) secondary PPD if cancer originates from the underlying colorectal or urinary tract neoplasm. Due to its rarity, only a few sporadic case reports have been published in the literature, and treatment methods are yet to be standardized. In light of this, we report two PPD cases with different etiopathogenesis and staging: one involved only the perianal skin without regional or metastatic disease, and was not accompanied by visceral adenocarcinomas although there was a previous history of sigmoid adenocarcinoma; the other was probably secondary to an anal canal tumor spreading with disseminated disease involving the perianal and perineum area with bilateral inguinal, pelvic lymph node, and liver metastasis. The treatment plans and the outcomes of both cases were necessarily different from each other.

## Introduction

Perianal Paget disease (PPD), a subset of extramammary Paget disease (EMPD), is an uncommon cutaneous neoplasm, which is often misdiagnosed and undertreated. Although clinical suspicion and punch biopsy are generally the basis of diagnosis, distinguishing between primary and secondary disease [1] is fundamental to carry out a correct assessment, provide an accurate treatment plan, and predict patient survival.

In primary PPD, where the disease arises as an intraepithelial neoplasm of the epidermis, surgical excision of the lesion is the standard of treatment with good results. On the other hand, secondary PPD requires a thorough review of systems and a full physical examination of the patients to detect an underlying tumor. In such cases, the recommended treatment and survival depend on the staging and location of the primitive tumor [2]. In secondary PPD, primitive cancer is usually hidden in the adjacent organs, mostly colorectal and genitourinary tracts, and has a poorer prognosis [3]. In this report, we present two cases of PPD with different etiopathogenesis, treatment methods, and outcomes.

## Case presentation

Case 1

A 73-year-old woman with a medical history of ischemic cardiomyopathy, congestive heart failure, non-insulin-treated type 2 diabetes, and chronic kidney disease was referred to our department. She had a right colon adenocarcinoma (pT3N1M0) and had undergone right hemicolectomy in another hospital three years ago, with no signs of neoplastic relapse. 

**Figure 1:**
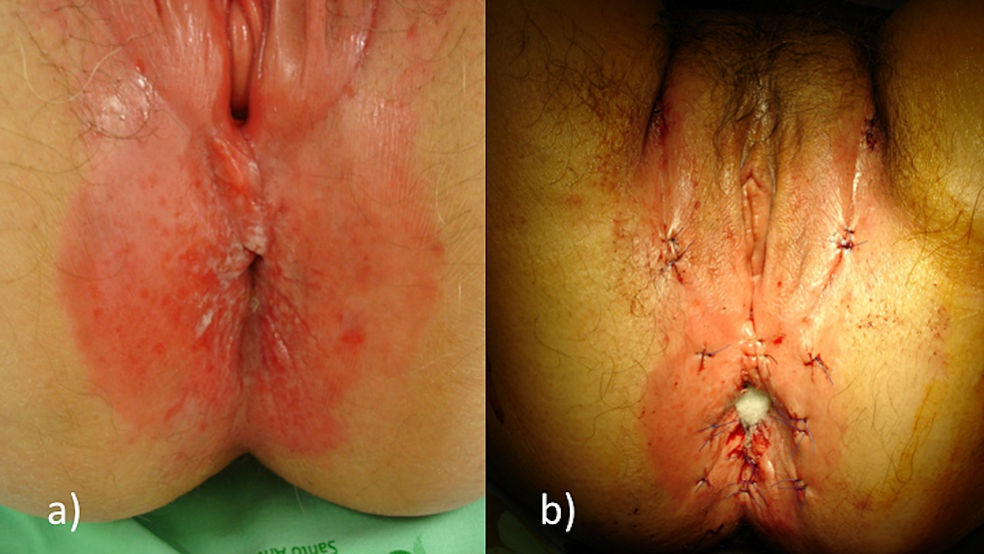
Physical examination of the patient a) Erythematous plaques of the perianal area, extending bilaterally from the groin area to the vulva and anus. b) Biopsies and skin tumor cells mapping

She had experienced complaints of anal pruritus for more than a year, with some transitory relief with topical ointments. In the previous month, she had experienced more severe complaints, with the appearance of erythema of the perineum. There were erythematous plaques on the perianal area on physical examination, extending bilaterally from the groin area to the vulva and anus, with no lymphadenopathies (Figure 1). 

**Figure 2:**
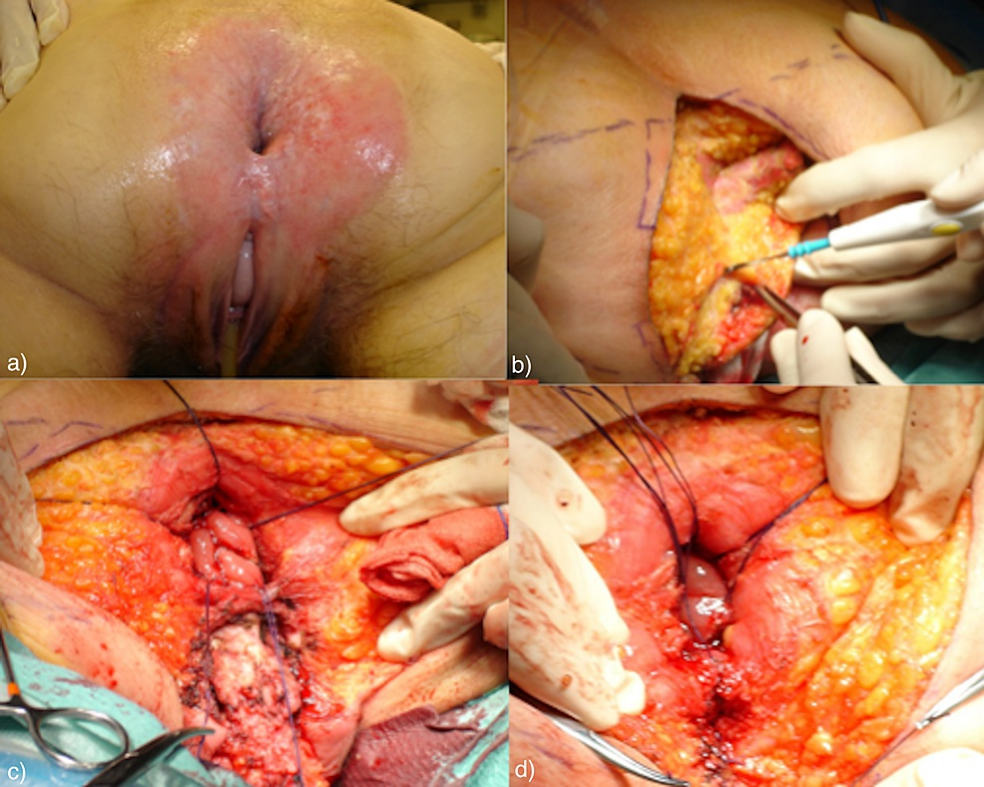
Surgical procedure: wide surgical excision with local flap reconstruction and diversion colostomy a) Patient in a jackknife position. b) Start of wide surgical excision. c) and d) Aspects of perineum after wide excision of perineal skin (anal canal referenced with wires) before the closure of the large defect with V-Y local flap

Skin biopsy was performed and revealed cytokeratin 7 (CK7)-positive, gross cystic disease fluid protein 15 (GCDFP-15)-positive, and CK20-negative cells, compatible with a diagnosis of primary PPD. Colonoscopy and thoracic, abdominal, and pelvic CT showed no significant alterations. The patient underwent wide surgical excision, local flap reconstruction, and a diversion colostomy (Figure 2).

**Figure 3:**
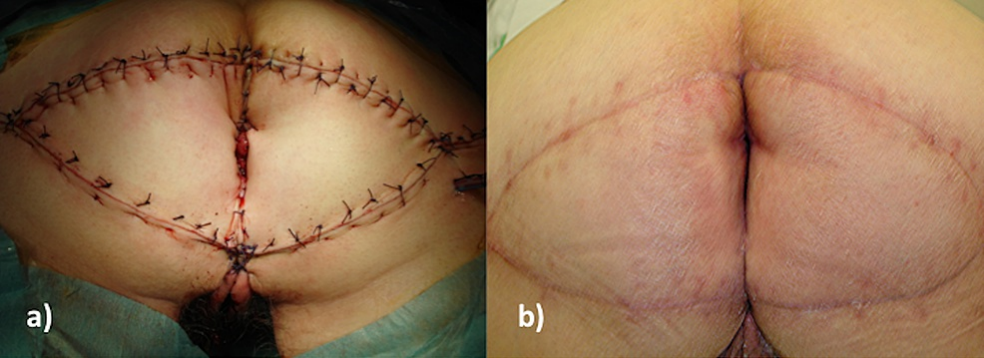
Surgical results a) Immediate outcome (at the end of surgery). b) Long-term outcome (12 months after surgery)

The surgical specimen showed a PPD restricted to the epidermis, with tumor-free resection margins. The postoperative period was uneventful. During the subsequent seven-year follow-up, the patient showed no signs of local relapse (Figure 3); she eventually passed away after that period due to her other comorbidities.

Case 2

A 73-year-old male presented to the emergency department (ED) complaining of fever accompanied by erythema and swelling involving the anus, perianal, pubic region, scrotum, and penis. The perianal skin lesions had been undergoing a year of evolution and were treated with topic ointments without a physician's counseling. The patient had a previous history of hypertension, chronic obstructive pulmonary disease, and smoking. 

**Figure 4:**
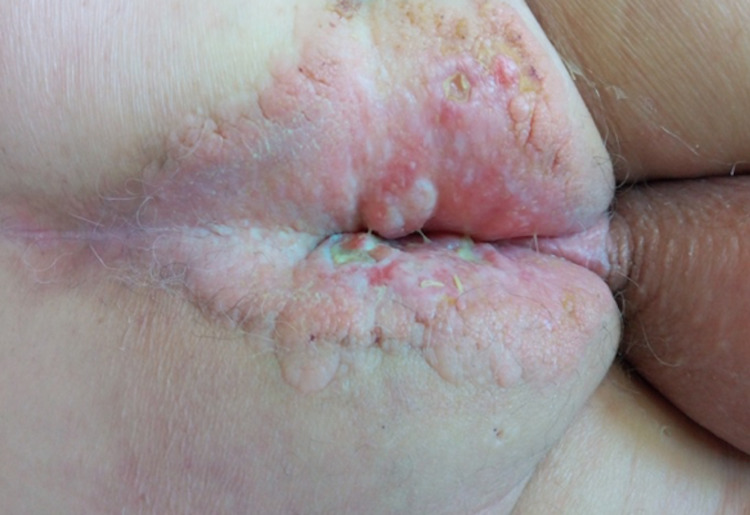
Extensive anogenital cellulitis with exuberant ulcerated lesions of the perianal skin

On physical examination, extensive anogenital cellulitis was confirmed with exuberant ulcerated lesions of the perianal skin (Figure 4). 

The patient was started on broad-spectrum antibiotic therapy. We performed biopsies given the lesions' suspicious character, including a polypoid lesion that protruded from the anal canal. The biopsies from perianal tissue, anus margin, and anal canal revealed adenocarcinoma with mucin-producing cells (CK7 and CK20+) and pagetoid spread, compatible with secondary PPD.

As there is a frequent association between PPD and underlying malignancy, further studies were performed. Colonoscopy only showed two hyperplastic polyps of the transverse and sigmoid colon. Cystoscopy and bladder lavage were performed to rule out the hypothesis of urogenital neoplasia.

**Figure 5:**
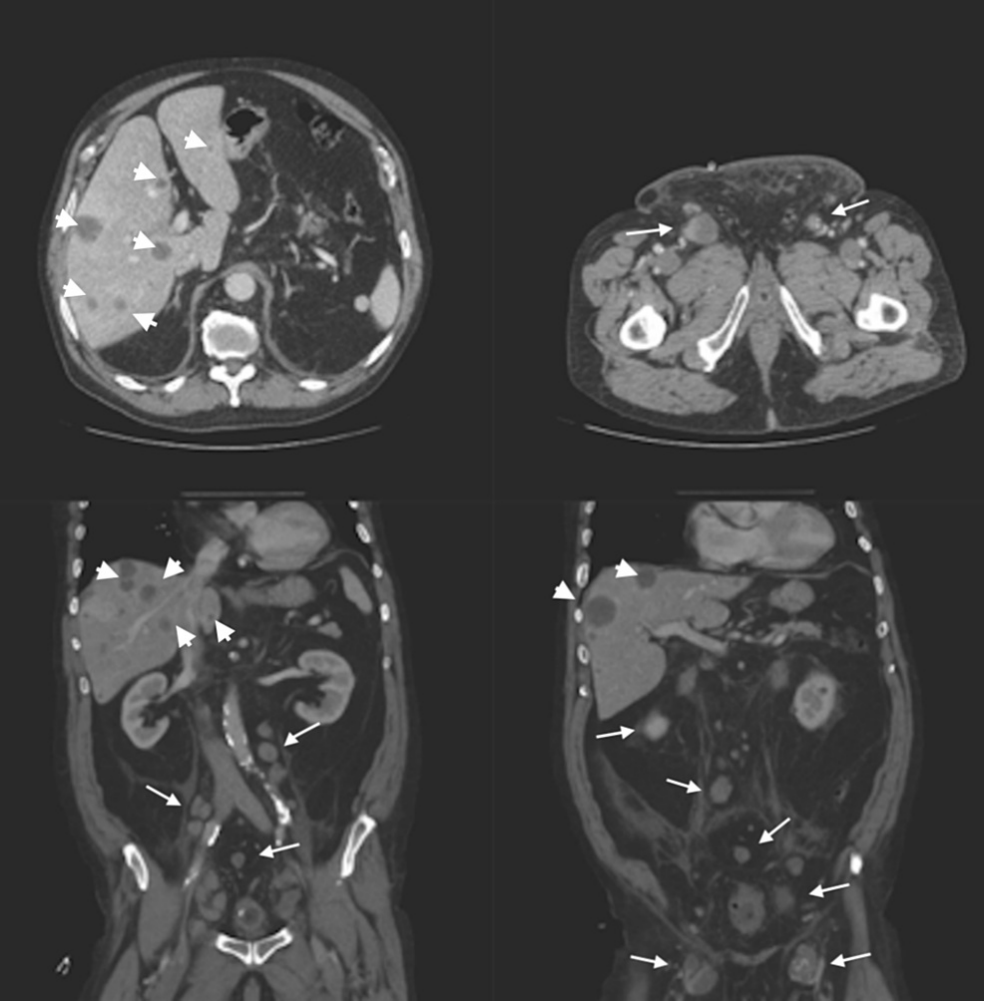
CT images The images show several hypodense nodules in both liver lobes suggestive of secondary lesions (thick arrows), and the presence of several retroperitoneal and groin pathological lymph nodes (thin arrows) CT: computed tomography

CT of the thorax, abdomen, and pelvis showed liver with several hypovascular lesions with rim enhancement, suggestive of secondary lesions, as well as the presence of several retroperitoneal pathological lymph nodes (Figure 5).

The patient's carcinoembryonic antigen (CEA) levels were elevated at 237.4 μg/L (normal range: <2.5 μg/L in an adult non-smoker and <5.0 μg/L in a smoker). A liver biopsy was performed, with pathology being compatible with liver metastasis (adenocarcinoma with signet-ring cells).

Due to the disease's advanced stage, the multidisciplinary oncology consultation decided to recommend the patient to palliative chemotherapy (capecitabine 1250 mg/m^2^ twice daily orally for two weeks followed by a one-week rest period in three-week cycles). The patient died six months after the diagnosis.

## Discussion

Paget disease, first described in 1874 by Sir James Page, is further classified into mammary and extramammary subgroups. EMPD is a rare neoplastic condition of the skin that occurs in the apocrine-rich locations such as the anoperineal (perianal, vulvar, and perineum), and axilla regions [4]. It accounts for 6.5% of all Paget diseases. The vulva is the predominant site of EMPD, accounting for up to 65% of cases [5]. Other common locations include the perianal area (20%) and male genitalia, including the scrotum or penis (14%).

PPD, a subset of EMPD, is an uncommon cutaneous neoplasm arising from the apocrine glands or underlying carcinoma in the perianal area. The true incidence of PPD is difficult to estimate due to its rarity, but it is known to represent less than 1% of all anal diseases and 1.3% of all cases of Paget disease [6]. It affects both men and women, particularly in their fifth to seventh decades of life.

This heterogeneous disease presents as multifocal, well-circumscribed erythematous or leukoplakic plaques or macules. Occasionally, hard nodules, palpable masses, or lymphadenopathy may be discovered, which should raise suspicion for invasive disease. The majority of the patients experience pruritus. However, burning, tenderness, and edema can also occur, as well as thickened plaque-like lesions with erythema or white scaly appearance in the skin [7]. A punch biopsy is necessary to confirm the diagnosis. Although several reports of PPD are available, there is still limited knowledge about its pathogenesis.

PPD is classified into two groups based on the main pathogenesis theory: primary and secondary PPD [8]. Primary PPD is epidermal stem cell-originated intraepithelial neoplasia in perianal skin and, similar to breast Paget disease, is not associated with other neoplasms. If the neoplasm penetrates the basement membrane and invades the stroma, lymphatic and other distant metastases may occur. Secondary PPD is an extracutaneous synchronous malignancy-originated neoplasia, and in it, the spreading of the remote carcinoma to the cutaneous lesion may be contiguous or not. Therefore, it is essential to evaluate whether patients carry thoroughly synchronous malignancy in remote sites or not. The diagnosis of secondary PPD is not always evident.

The primary disease is the most common form, although the secondary form accounts for about 40% of the cases. The key factors related to diagnosis are clinical suspicion and a biopsy that confirms Paget cells' presence. This procedure is usually performed in both groups. The presence of Paget cells in the biopsy, which are large tumor cells with pale clear cytoplasm and large hyperchromatic nuclei, is the key for histologic diagnosis. On immunohistochemistry, Paget cells display strong cytoplasmic CEA positivity.

To differentiate primary PPD from secondary PPD, primary PPD usually displays positive expressions of CK7 and GCDFP-15, a sensitive marker for apocrine differentiation and CD-15 (Leu-M1) and lysozyme, with a lack of CK20 expression, equatable with sweat gland differentiation [9]. On the contrary, secondary PPD usually displays positive CK7 and CK20 with negative GCDFP-15, pointing to endodermal differentiation [10,11].

In our report, we discussed both primary and secondary presentations. We believe that the first case represented a primary PPD, even though the patient had a previous history of sigmoid adenocarcinoma. The second case was possibly a secondary PPD originating from an adenocarcinoma of the anal canal with pagetoid spreading. Primary adenocarcinomas of the anal canal are uncommon. Most anal canal carcinomas are squamous cell carcinomas (SCC). Only 20% of anal canal tumors are adenocarcinomas of anal gland origin. Pagetoid spread for perianal skin is rare but possible. 

In our cases, histologic findings enabled us to differentiate primary PPD from secondary PPD. In general, patients with primary PPD have a good prognosis with a five-year overall survival rate of 75-95%. Although PPD is usually intraepithelial, it can progress to invasive disease with metastases. So, regardless of whether it is a primary or secondary disease, PPD can be diagnosed at several stages of the disease. The treatment mainly depends on the tumor stage than on the etiopathogenesis. If the disease is located, surgical resection is the most common treatment modality with curative intent. Radiotherapy and/or chemotherapy are generally opted for metastatic disease, although there are reports of its use as neoadjuvant or adjuvant therapy [12]. Radiotherapy can be used for symptom control or as an alternative to surgery in inoperable cases. It has also been used in neoadjuvant settings before surgery in patients with dermal invasion to achieve better tumor control, namely by prophylactic lymph node irradiation.

The use of concurrent chemotherapy has also been described. Adjuvant radiotherapy has been recommended, especially in multifocal disease, lymph node metastasis, positive surgical margins, and associated malignancies. Although there is no established standard regimen, the steps that we undertook, which are described below, are the therapeutic rules that the physicians must follow. Since there was no previous tumor relapse in the first case, a surgical skin-wide resection enabled us to perform surgery with curative intention and attain a good outcome. If the primary tumor had been present in the first case, its surgery would have been mandatory alongside the skin-wide resection. In this case, we performed an intraoperative frozen scouting biopsy (mapping biopsy) to determine the resection margin. Scouting biopsy has been considered to be a proper preoperative method to evaluate the disease extent in the perianal skin and within the anal canal [13]. No consensus on the extent of the resection margin exists. Recent studies on the surgical treatment of PPD have shown recurrence rates of 33-60% with a lateral resection margin of 1-3 cm and a deep margin of 0.5 cm [14]. We followed these rules and performed a wide excision of the rectal mucosa up to 2 cm above the dentate line without a relapse history. Reconstruction using V-Y advancement flap and wound infection prevention by fecal diversion led to an excellent cosmetic result without morbidity.

Other options were indeed possible instead of the wide-skin resection, such as topical chemotherapy, or laser or radiotherapy, but these have shown varying results [15,16]. In our opinion, these therapeutic alternatives should be employed only for patients for whom surgery is not possible. On the other hand, the second case was probably a secondary PPD (adenocarcinoma of the anal canal with pagetoid spread) presented at an advanced stage with metastatic disease in superficial inguinal, deep pelvic, and superior retroperitoneal lymphatic nodes (due to the mixed lymphatic drainage of the anal canal) and in the liver, which led to palliative treatment. If the disease stage had been III and not IV, abdominoperineal resection and aggressive chemoradiation could have been the options, but those are associated with poor survival.

When we analyze the prognosis impact of etiopathogenesis, the literature predominantly emphasizes that secondary disease tends to be more aggressive than primary PPD [17]. It is usually due to the presence of underlying malignancy and the higher possibility of metastases. In our cases, the first patient had a previous history of gastrointestinal cancer, which raised the hypothesis of secondary PPD, although histologic features supported the assumption of primary PPD. In this case, the disease had no invasive features and remained free of recurrence during the subsequent seven-year follow-up. Also, we considered that secondary PPD was likely but presenting at an advanced stage and with a poor prognosis in the second case. However, regardless of whether the disease is primary or secondary in nature, in our experience, the PPD stage and the possibility of performing surgery are the main factors that dictate the prognosis.

## Conclusions

PPD has a broad spectrum of clinical presentations, sometimes being associated with underlying malignancies, particularly in the gastrointestinal tract. As it is a rare form of tumor, it is sometimes misdiagnosed and not thoroughly investigated. A biopsy lesion is crucial for the diagnosis. The patient should be fully studied with endoscopic and tomographic exams to evaluate for synchronous tumors. Correct studies and proper treatment are crucial for ensuring a good prognosis.
